# Early Career Psychiatrists in Education and Academia in Europe – Challenges and Ways Forward

**DOI:** 10.1192/j.eurpsy.2022.205

**Published:** 2022-09-01

**Authors:** F. Baessler

**Affiliations:** Centre for Psychosocial Medicine, Department Of General Internal And Psychosomatic Medicine, Heidelberg, Germany

## Abstract

Academic research and publications can serve as important drivers of career development for early-career psychiatrists. However, the current focus on clinical teaching during the postgraduate program leaves much less room for scholastic training for young professionals. Combined with a lack of standardized European curricula despite automatically recognized qualifications in European Union countries, early-career psychiatrists encounter challenging job prospects across Europe, often faced with the hard choice of pursuing careers in clinical practice or opt for academia. In recent years, academia has attracted more and more young psychiatrists eager to contribute as researchers, teachers and/or administrators within academic and higher education institutions. What are the challenges they face and how they can overcome them to combine clinical work, teaching and research? The component of teaching and research varies widely in European psychiatry training and mostly early-career psychiatrists are unaware about the importance of publications, formal qualifications, stipends, international experiences, participating in studies, writing grant applications, etc, for their academic career. Often, they are not clearly informed about the advantages/disadvantages of pursuing an academic career and learn at a later stage about the important steps towards a successful career combining clinical work, research and teaching. In this session, we will share a brief insight into the challenges of academia and the possible ways forward with some real-life experiences of the speaker, who is leading several interdisciplinary research projects at the Heidelberg University Hospital along with handling patients and family at the same time. An interactive discussion and exchange of knowledge is desired.

**Disclosure:**

No significant relationships.

